# Asymmetrical characteristics of emotional responses to pictures and sounds: Evidence from pupillometry

**DOI:** 10.1371/journal.pone.0230775

**Published:** 2020-04-06

**Authors:** Satoshi Nakakoga, Hiroshi Higashi, Junya Muramatsu, Shigeki Nakauchi, Tetsuto Minami

**Affiliations:** 1 Department of Computer Science and Engineering, Toyohashi University of Technology, Toyohashi, Aichi Japan; 2 Graduate School of Informatics, Kyoto University, Sakyo-ku, Kyoto, Japan; 3 Electronics Control System Development Div, Body Electronics System Development Dept, TOYOTA MOTOR CORPORATION, Toyota, Aichi, Japan; 4 Electronics-Inspired Interdisciplinary Research Institute, Toyohashi University of Technology, Toyohashi, Aichi, Japan; Dalhousie University, CANADA

## Abstract

In daily life, our emotions are often elicited by a multimodal environment, mainly visual and auditory stimuli. Therefore, it is crucial to investigate the symmetrical characteristics of emotional responses to pictures and sounds. In this study, we aimed to elucidate the relationship of attentional states to emotional unimodal stimuli (pictures or sounds) and emotional responses by measuring the pupil diameter, which reflects the emotional arousal associated with increased sympathetic activity. Our hypothesis was that the emotional responses to both the image and sound stimuli are symmetrical: emotion might be suppressed when attentional resources are allocated to another stimulus of the same modality as the emotional stimulus—such as a dot presented at the same time as an emotional image, and a beep sound presented at the same time as an emotional sound. In our two experiments, data for 24 participants were analyzed for a pupillary response. In experiment 1, we investigated the relationship of the attentional state with emotional visual stimuli (International Affective Picture System) and emotional responses by using pupillometry. We set four task conditions to modulate the attentional state (emotional task, no task, visual detection task, and auditory detection task). We observed that the velocity of pupillary dilation was faster during the presentation of emotionally arousing pictures compared to that of neutral ones, regardless of the valence of the pictures. Importantly, this effect was not dependent on the task condition. In experiment 2, we investigated the relationship of the attentional state with emotional auditory sounds (International Affective Digitized Sounds) and emotional responses. We observed a trend towards a significant interaction between the stimulus and the task conditions with regard to the velocity of pupillary dilation. In the emotional and auditory detection tasks, the velocity of pupillary dilation was faster with positive and neutral sounds than negative sounds. However, there were no significant differences between the no task and visual detection task conditions. Taken together, the current data reveal that different pupillary responses were elicited to emotional visual and auditory stimuli, at least in the point that there is no attentional effect to emotional responses to visual stimuli, despite both experiments being sufficiently controlled to be of symmetrical experimental design.

## Introduction

Emotions have been extensively investigated in fields such as cognitive psychology and neuroscience. Research on emotional processing has almost exclusively used a collection of emotional pictures known as the International Affective Picture System (IAPS; [1]) and a collection of emotional sounds known as the International Affective Digitized Sounds (IADS; [2]) as the stimulus material. However, an issue with past studies of emotion research is that they have typically only investigated unimodal cues [3].

Most studies on audiovisual integration, such as the McGurk effect, have reported that the influence of visual stimuli in these interactions is dominant over that of auditory stimuli [4,5]. The fMRI study by Anders et al. revealed a higher differential activation in the amygdala during exposure to the emotional pictures from the IAPS compared to the emotional sounds from the IADS [6]. Another study suggested that differential brain activation in response to the emotional pictures and sounds may not be due to the differences in the emotional processing; rather, it may be due to methodological differences and different stimulus characteristics [3]. Although emotional responses to sounds are weaker [7] and occur later [8], the processing of emotional sounds and pictures is comparable based on behavioral, physiological, and electrophysiological reactions [9,10]. However, it is not clear whether the characteristics of emotional responses to pictures and sounds are symmetric in every situation. To assess this, the present study investigated the relationship of attentional states to emotional unimodal stimuli (pictures or sounds) based on the hypothesis that emotional responses elicited by pictures and sounds are asymmetric. The subjective evaluation methods of emotional stimuli, including the Self-Assessment Manikin (SAM; [11]), which is a method for the subjective rating of the dimensions of valence and arousal, do not reflect the emotional state when paying attention to emotional stimuli (pictures or sounds), because it is conducted following a stimulus presentation. Therefore, in the present study, we measured the pupil diameter, which reflects the emotional arousal associated with increased sympathetic activity, as a physiological indicator to record the emotional state during the stimulus presentation.

Pupil dilation is controlled by the level of activation of the locus coeruleus (LC), which has an important role in cognitive function and arousal [12,13]. Therefore, a pupillary response is considered a physiological indicator of mental activity [14–16]. Studies on the association between pupillary changes and emotional responses have reported that pupil diameter was dilated as a results of increase in arousal following presentation of an emotional stimuli such as the IAPS pictures and the IADS sounds [17–24]. Snowden et al. reported that pupil dilation in response to emotional pictures was not affected by actively naming the emotion of the stimuli compared to passive viewing [22]. In the study by Kinner et al, pupillary responses were measured while participants controlled their emotional states with cognitive emotion regulation (e.g., reappraisal, distraction) during the presentation of the IAPS pictures. When participants performed emotion regulation, pupil dilation was decreased compared to the passive viewing of the negative pictures. This suggested that pupillary response could be an indication of the success or failure of the emotion regulation [24].

This study investigated the relationship of attentional states with emotional unimodal stimuli (pictures or sounds) using pupillometry in two experiments. Typical examples of the distraction task during a presentation of emotional stimuli are continuous target counting task by Schupp et al. [25,26]. However, these tasks are not suitable for pupillometry, which is sensitive to the change of brightness and requires an appropriate interval between trials. Thus, in addition to the 2 tasks in Snowden’s experiment (emotional task, no task), we added two new tasks to capture visual or auditory attention (visual detection task, and auditory detection task). In these tasks, a visual or auditory target (a dot or beep sound) was presented in random trials. However, the trials in which the target appears in these tasks cannot be compared with other tasks due to the difference in screen brightness or sound frequency. Accordingly, we attempted to control and equalize attentional states in all task conditions by analyzing the pupillary response of the trials without the target. In addition, the stimulus conditions of each emotional stimulus in this study were categorized based on subjective evaluation in each participants because objective and subjective assessments of the IAPS and IADS are not always consistent, and there is an individual difference [27,28]. Our hypothesis is that the emotional responses to both the images and the sounds stimuli might be suppressed when attention was paid to the target stimuli of the same modality as to emotional stimuli. Although there is evidence for this emotional suppression effect for vision [25], it is still not completely understood whether the same effects occur for audition. Therefore, we investigated whether emotional responses to visual and auditory stimuli had symmetrical properties by comparing the pupillary response depending on the emotional state in each task condition.

## Materials and methods

### Participants

Twenty-six healthy participants (10 females) participated in Experiment 1 (mean age = 22.88 years, S.D = 2.65), and 24 healthy participants (8 females) participated in Experiment 2 (mean age = 22.00 years, S.D. = 1.53). Six of the 24 participants from Experiment 2 also participated in Experiment 1 approximately 1 month before Experiment 2. They were recruited through e-mail by us. Their performance did not differ from the other participants. All participants are students or staff in Toyohashi University of Technology. They had normal or corrected-to-normal vision, normal hearing based on self-reports and they were not informed of the purpose of the study. Two participants from Experiment 1 were excluded from the analyses due to artifacts such as eye blinks and missing data. A power analysis using G*Power software (G*power 3.1; [29]) indicated that a sample size of 24 would achieve 80% power for a repeated measures design, given a medium effect size (*f* = 0.25) and α = 0.05. All participants provided written informed consent. The experimental procedure received approval from the Committee for Human Research at Toyohashi University of Technology. The experiments were conducted strictly in accordance with the approved guidelines of the committee.

### Stimuli

For Experiment 1, pictures were selected from the IAPS based on the normative ratings [1]. Sets of 20 positive pictures (valence: mean = 7.29, S.D. = 0.40; arousal: mean = 6.20, S.D. = 0.84), 20 negative pictures (valence: mean = 2.77, S.D. = 0.57; arousal: mean = 6.20, S.D. = 0.55), and 20 neutral pictures (valence: mean = 5.06, S.D. = 0.26; arousal: mean = 4.24, S.D. = 0.98) were created. All pictures were in landscape orientation (19.0 × 14.3 degrees of visual angle) and were displayed in grayscale. Mean luminance of the selected pictures was matched using the MATLAB R2016a (MathWork Inc.) SHINE toolbox [30]. In order to match the level of luminance prior to the picture onset, the luminance of the gray background was controlled with the mean luminance computed across all pictures.

For Experiment 2, sounds were selected from the IADS based on the normative ratings [2]. Sets of 20 positive sounds (valence: mean = 7.22, S.D. = 0.55; arousal: mean = 6.30, S.D. = 0.85), 20 negative sounds (valence: mean = 2.80, S.D. = 0.45; arousal: mean = 6.28, S.D. = 0.63), and 20 neutral sounds (valence: mean = 4.99, S.D. = 0.40; arousal: mean = 4.32, S.D. = 0.61) were created. The stimuli were selected for comparable valence and arousal ratings between pleasant and unpleasant stimuli and between Experiment 1 and Experiment 2. All sounds were normalized for peak amplitude to standardize their loudness. In order to match the level of luminance between Experiment 1 and 2, the luminance of the gray background was controlled with the mean luminance computed across all pictures in Experiment 1.

### Procedure

Experiment 1 and Experiment 2 were conducted separately and on different days. In both experiments, after participants received an explanation of the experimental procedure, they signed an informed consent form. Participants were then seated in comfortable chairs with their chins fixed at a viewing distance of 60 cm, in a shielded dark room. Visual stimuli were displayed on a calibrated 24-inch LCD monitor (ViewPixx3D, VPixx Technologies). Auditory stimuli were presented through headphones (SoundTrue around-ear headphones, BOSE). All experiments were developed in Windows 10 and executed in MATLAB 2014b (MathWork Inc.) using Psychtoolbox 3 [31–33].

#### Experiment 1

**Fig 1:**
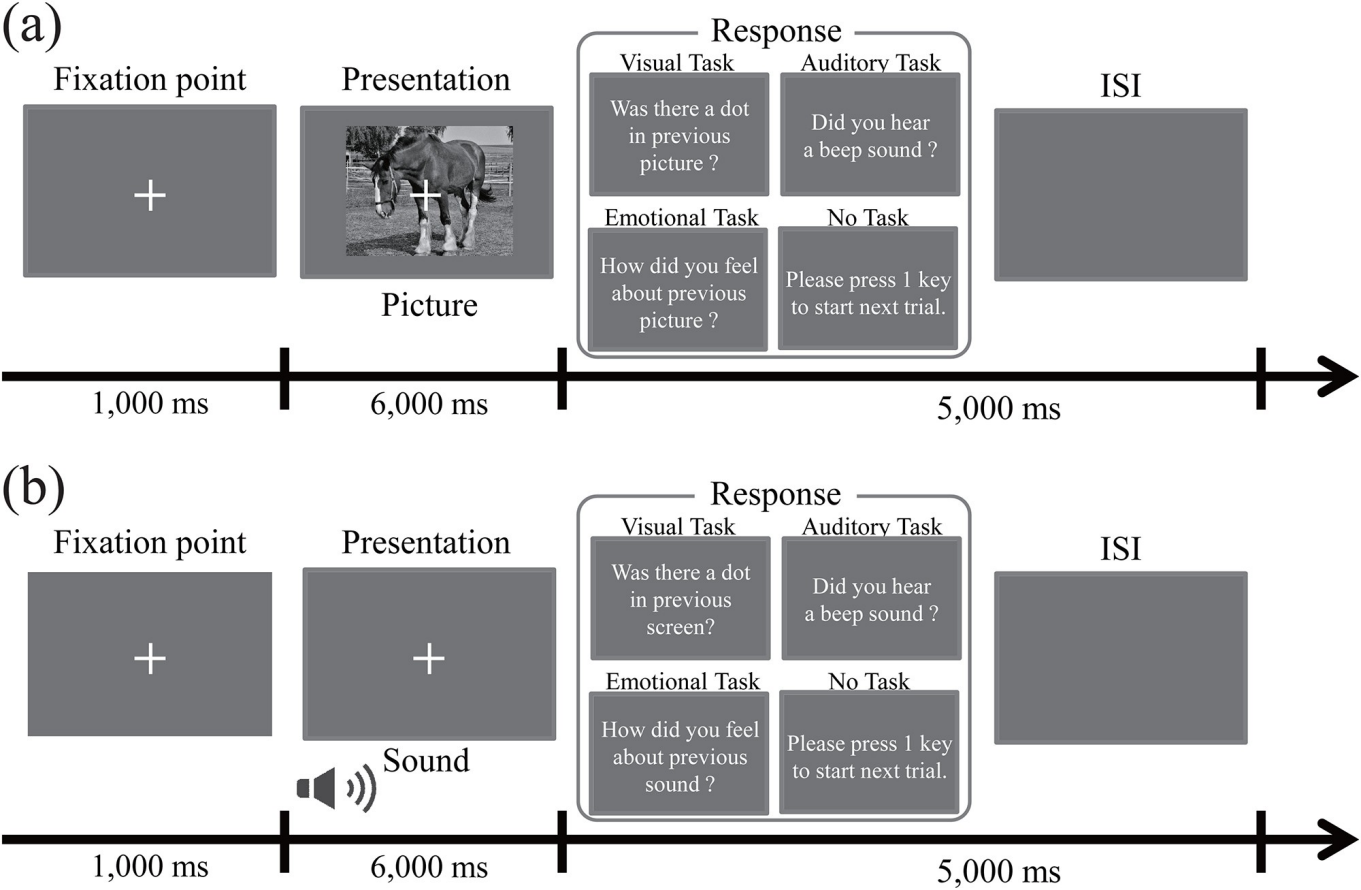
Experimental procedure. **(a)** Protocol for Experiment 1. In each trial, the fixation point was presented for 1,000 ms. The IAPS picture was then presented for 6,000 ms. Each trial was separated by a response period and an inter-stimulus interval (ISI) of 5,000 ms in total. Participants responded to the task in each block following the stimulus presentation using the keypad. **(b)** Protocol for Experiment 2. The paradigm (presentation times, participants’ tasks, and the number of trials) was identical with that in Experiment 1. However, IADS sounds and not IAPS pictures were used.

Fig 1a shows the protocol for Experiment 1. The experiment consisted of 60 IAPS pictures and was conducted over four blocks. In these four blocks, the 60 stimuli were identical, and each stimulus was presented only once within each block in random order. In each trial, a fixation point was presented for 1,000 ms prior to the presentation of the stimulus. Each IAPS picture was presented for 6,000 ms. The participants were instructed to fixate on a central point during the presentation of the fixation point and stimulus. They responded to the task in each block by pressing a key after stimulus presentation. The tasks in each block differed as follows: (1) Emotional task: the subjective emotional evaluation of presented pictures (positive, negative, or neutral); (2) No task: only pressing the key as a control condition; (3) Visual task: the visual detection of a dot, which was added to the IAPS picture during stimulus presentation at random (appearance or no appearance); (4) Auditory task: the auditory detection of a beep sound, which was emitted during stimulus presentation through a set of headphones at random (appearance or no appearance). In the visual task block, the target dot in random trials was added to a picture for 100 ms at random intervals during the stimulus presentation (somewhere between 1,000 ms and 4,500 ms after stimulus presentation). The target dot was a circle of 3 pixels radius, and it was superimposed at a random position onto the IAPS pictures. In the auditory task block, the target beep sound in random trials was presented for 300 ms at random intervals during stimulus presentation (somewhere between 1,000 ms and 4,500 ms after stimulus presentation). The presentation of the target dot or beep sound accounted for 20% of all trials in each block. However, these trials could not be analyzed for pupillary response because of differences in experimental conditions compared to the other trials. Therefore, the number of trials in the visual and auditory tasks was 75, in order to equalize the number of analyzable trials in all task conditions. In order to equalize the accuracies of visual and auditory tasks at 75% in the preliminary experiment, the target dot (area and color) and target beep sound (frequency and volume) were modulated. Participants had a maximum of 5,000 ms to respond, after which the next trial started automatically. Participants rested for at least 5 min between each block. The order of the tasks was counterbalanced across the participants.

#### Experiment 2

Fig 1b shows the protocol for Experiment 2. The experiment consisted of 60 IADS sounds and was conducted over four blocks. The presentation times, participants’ tasks, and the number of trials in Experiment 2 were identical to those in Experiment 1. However, instead of the IAPS pictures in Experiment 1, IADS sounds were presented as stimuli. In the visual task block, the target dot was a circle of 3 pixels radius, and it was presented at a random position within the same range as Experiment 1. In the auditory task block, the target beep sound was synthesized as part of the IADS sound. In order to equalize the accuracies of the visual and auditory tasks at 75% in a preliminary experiment, the target dot (area and color) and the target beep sound (frequency and volume) were modulated with different parameters to those in Experiment 1 as the IADS sounds were presented instead of the IAPS pictures.

### Pupillometry

Pupil diameter and eye movement during the presentation of the fixation point and stimulus were recorded using an eye-tracking system (Eyelink 1000, SR Research), at the sampling rate of 500 Hz. The eye-tracking system was desk mounted and used infrared video-based tracking technology. The movement of the participant’s left eye was recorded using an infrared video camera at the resolution of up to 0.1°. A 9-point calibration and validation were performed before the start of each block to ensure that the participant’s eyes were correctly tracked by the eye-tracking system.

### Data analysis

#### Behavioral data

The rates at which participants’ responses to the stimuli in the emotional task matched the valence of the stimuli. They were computed for both experiments and analyzed with a one-way repeated-measures analysis of variance (ANOVA) for each stimulus condition (positive, negative, and neutral) as factors. The level of statistical significance was set at *p* < 0.05 for all analyses. Pairwise comparisons for the main effects were corrected for multiple comparisons using the Bonferroni method. Effect sizes (*partial η*^*2*^) were determined for the ANOVA. In addition, the detection rates of the target dots or beep sounds for visual or auditory tasks were computed, and a paired t-test was used to compare the detection rates between visual and auditory tasks in each experiment.

#### Pupillary data

The pupillary data were prepared and analyzed using MATLAB 2017b (MathWork Inc.). The trials in which the target dot or beep sound appeared in the visual or auditory tasks were rejected from the pupillary data analysis. In both experiments, some erotic images and sounds were included in positive stimuli. Several studies have shown gender differences in emotional responses to erotic images [34,35]. In fact, the results of emotion classification in the emotional task indicated that there were many erotic stimuli that the male participants responded to positively, whereas the female participants responded negatively rather than positively. Therefore, pupillary data in each trial was classified into three conditions (positive, negative, and neutral) according to the participants’ responses in an emotional task rather than the value of the emotional valence based on the normative ratings in IAPS and IADS. Due to this classification method, the trials analyzed for pupillary response in each emotion condition were in the range of 10 to 40. The eye blinks were interpolated using cubic-spline interpolation [36]. The trials containing artifacts were removed using the principal component analysis (PCA) and the peak change of the velocity of the pupil response.

The PCA was performed to reject the trials including potential contaminant using all pupillary time course data in each experiment as the input data. The threshold for detection of the peak change of the velocity was defined based on Mathôt’ et al.’s research [36]. It was used to reject the trials which were not interpolated the eye blinks and the loss for a sustained period by cubic-spline interpolation. Based on these methods for artifact removal, 4.36 trials in Experiment 1 and 3.83 trials in Experiment 2 for each condition, on average, were excluded from the analysis. Two participants from Experiment 1 were excluded because all trials in one of the experimental conditions (20 trials) or more than 75% of one of the task conditions (60 trials) were rejected due to artifacts. After the artifact removals, the pupillary data for the analyses in each emotion condition were in the range of 9 to 39.

In the time course analysis, each trial data was down-sampled to 50 Hz and subsequently each data point ± four sampling points were smoothed. Next, the pupillary area data were converted to diameter data in accordance with the method proposed by Hayes and Petrov [37] because the pupil size was generated by the device in arbitrary units. The baseline pupil size was computed as an average of the data collected prior to the stimulus onset (picture or sound presentation), from -200 ms to 0 ms (presentation onset). The baseline-corrected average pupil sizes for the presentation period of 0 ms to 5,800 ms in both experiments were compared to each condition [38].

Based on Kinner’s research, a gradient in Experiment 1 was calculated between the minimum pupil diameter (between 0 and 1,000 ms after the picture onset) and maximum pupil diameter (between 1,000 and 2,000 ms after the picture onset) [24]. Similarly, a gradient in Experiment 2 was calculated between the maximum pupil diameter (between 0 and 1,000 ms after the sound onset) and maximum pupil diameter (between 1,000 and 2,000 ms after the sound onset). Pupillary responses in Experiment 1 constricted from the picture presentation due to the light reflex, whereas pupillary responses in Experiment 2 dilated due to the sound stimuli and no change in the screen. The gradient values were used as physiological indexes in this study. They were computed and analyzed using a repeated measures ANOVA for each stimulus condition (positive, negative, and neutral). Two-way ANOVAs were performed using the pupillary gradient in each stimulus condition (positive, negative, and neutral) and the task conditions (emotional task, no task, visual task, and auditory task) as factors. The level of statistical significance was set at *p* < 0.05 for all analyses. The pairwise comparisons for the main effects were corrected for multiple comparisons using the MSRB (Modified Sequentially Rejective Bonferroni) procedure [39]. Effect sizes (*partial η*^*2*^) were determined for the ANOVA.

## Results

### Behavioral response

**Fig 2:**
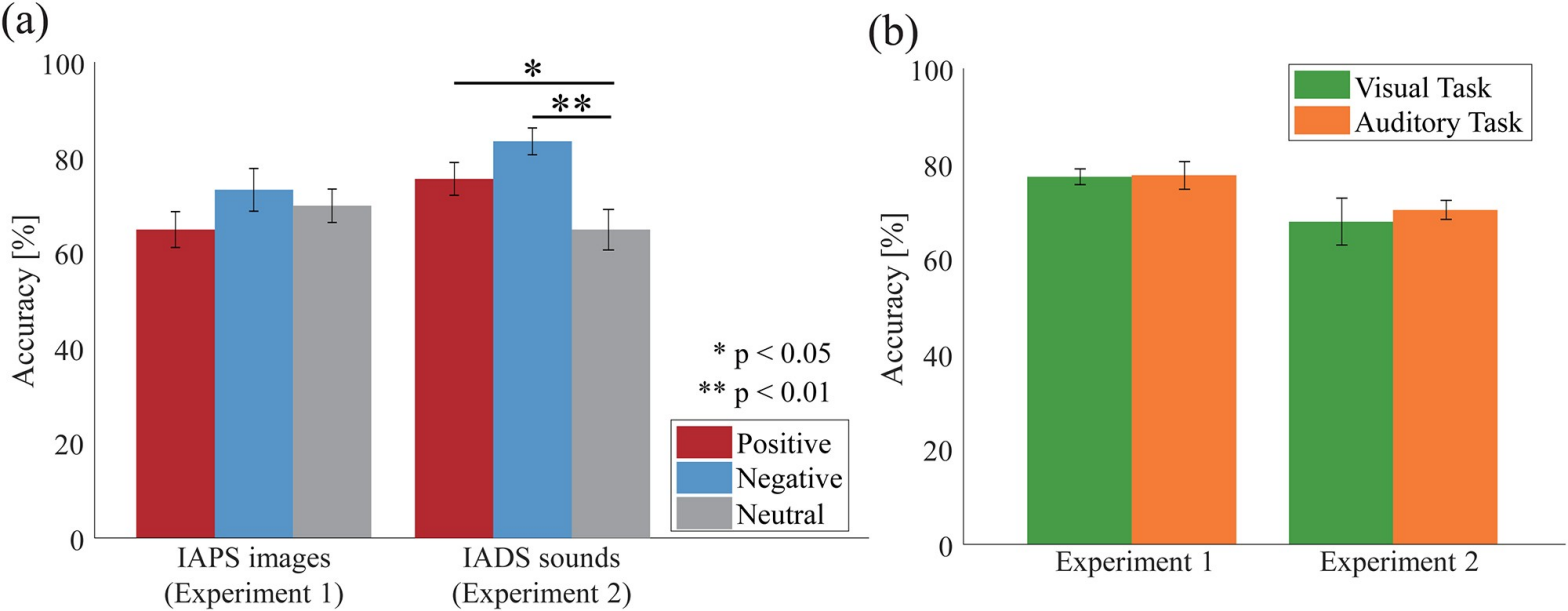
Behavioral results. (a) Mean rates at which the participants’ responses to the pictures and sounds in the emotional task matched the valence of the pictures and sounds in Experiment 1 and 2. (b) Detection rates of the target dot and beep sound for the visual task and the auditory task, respectively, in both experiments. The error bars indicate the standard error of the mean across the participants. Asterisks in (a) indicate a significant difference based on multiple comparisons for the main effect of stimulus; * p < 0.05, ** p < 0.01.

Fig 2a shows the rates at which participants’ responses to the pictures and sounds in the emotional task of Experiment 1 and 2 matches the valence of the pictures and sounds. ANOVA revealed no main effect of the stimulus condition in Experiment 1 [*F*(2, 46) = 1.158; *p* = 0.323; partial *η*^*2*^ = 0.048]. In addition, we analyzed these rates with a Bayesian repeated measures ANOVA using the statistical software JASP (httos://jasp-stats.org/). We concluded that there is no main effect of the stimulus condition in Experiment 1. We found a BF_10_ smaller than 0.33 in the main effect of the stimulus (*BF*_*10*_ = 0.323). Thus, we have evidence in support of the null hypothesis [40] that there is no main effect of the stimulus. These results indicate that participants’ emotions elicited by IAPS pictures in Experiment 1 were impartial. On the other hand, there is a significant main effect of the stimulus condition in Experiment 2 [*F*(2, 46) = 7.923; *p* = 0.001; partial *η2* = 0.256; *BF*_*10*_ = 64.401]. Planned comparisons revealed that the rate at which responses matched the valence of the negative sounds was significantly higher than that for the neutral sounds (*p* = 0.003) and showed the trend to be significantly higher than that for the positive sounds, although it did not reach statistical significance (*p* = 0.061). Moreover, the rate at which responses matched the valence of the positive sounds was significantly higher than that for the neutral sounds (*p* = 0.048). These results indicate that negative emotions, rather than positive or neutral emotions, were most strongly elicited by the IADS sounds in Experiment 2.

Fig 2b shows the detection rates of the target dot and beep sound for the visual and auditory tasks in both experiments. T-tests revealed no significant differences for task conditions in both experiments (Experiment 1: *p* = 0.940, Experiment 2: *p* = 0.637). In addition, we analyzed these rates with Bayesian paired sample t-tests. Bayesian t-tests showed moderate evidences for the null hypothesis that there is no difference in the detection rates between visual and auditory tasks in both experiments (Experiment 1: *BF*_*10*_ = 0.215, Experiment 2: *BF*_*10*_ = 0.238). These results indicate that the difficulties of the visual and auditory tasks in both experiments did not differ.

### Pupillary response

**Fig 3:**
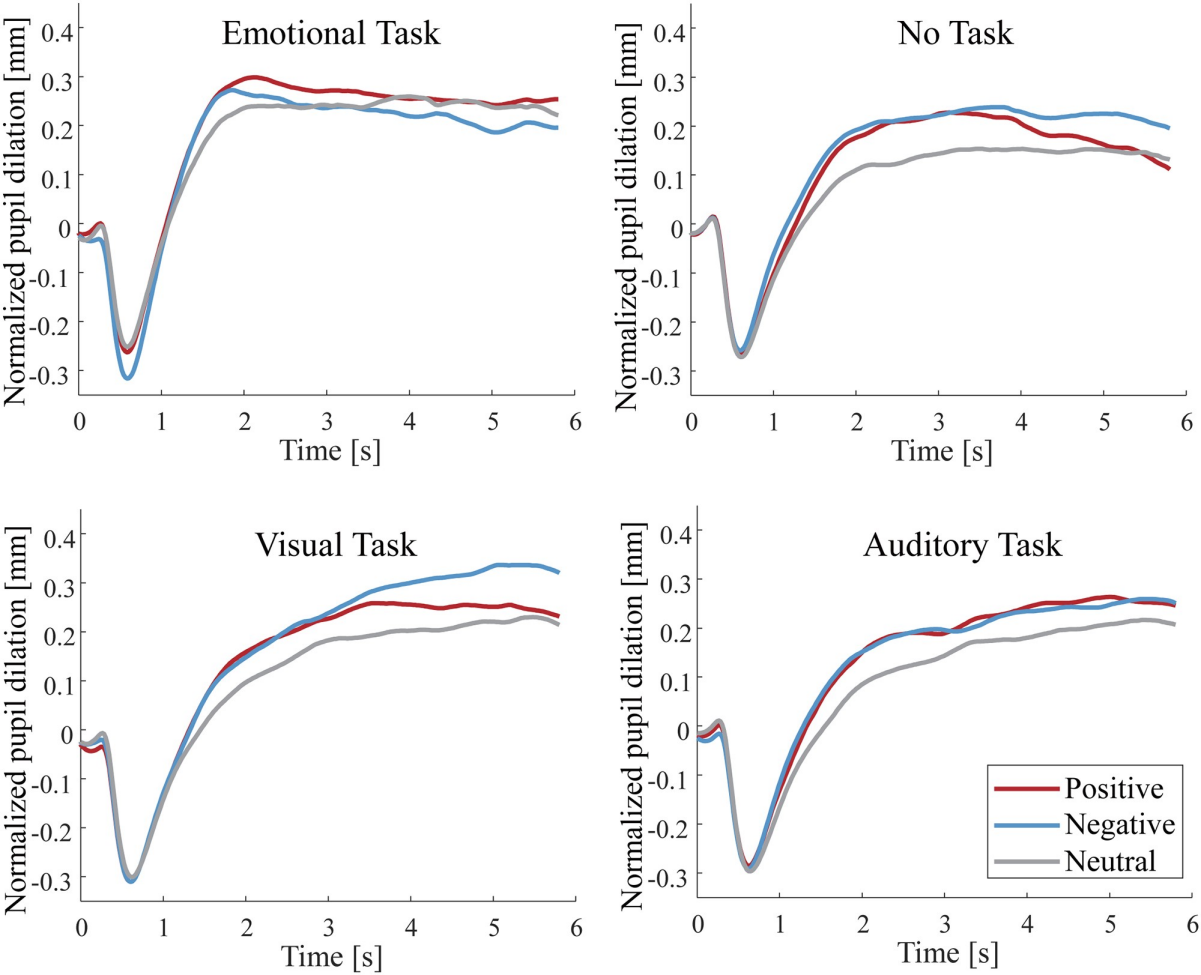
The grand-averaged time course of pupillary responses to the IAPS pictures (positive, negative, and neutral) in the task conditions during the stimulus presentation in Experiment 1. The horizontal axis indicates the time (s), while the vertical axis indicates the grand-averaged change in pupil dilation from baseline (−200 ms to 0 ms).

**Fig 4:**
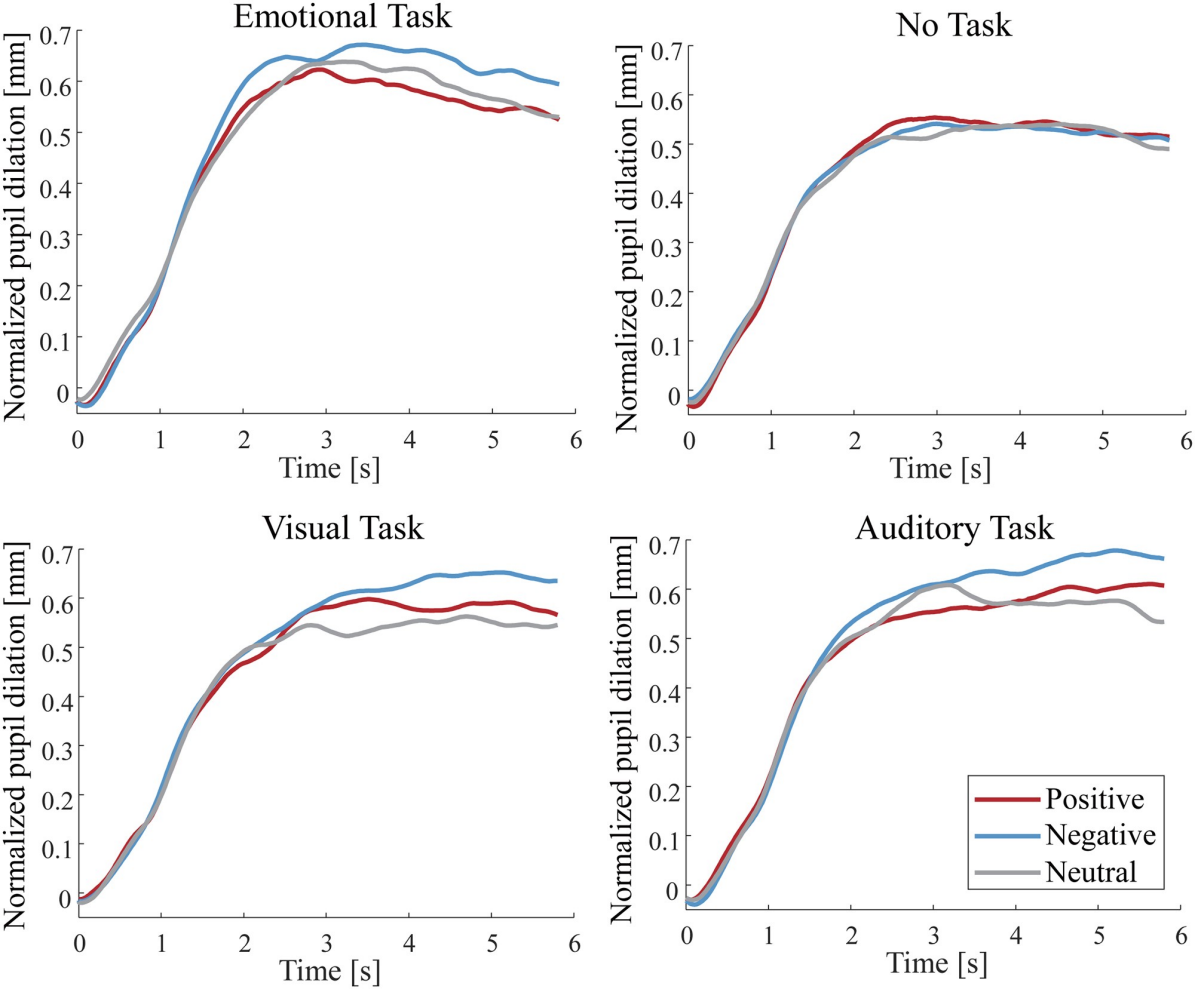
The grand-averaged time course of pupillary responses to the IADS sounds (positive, negative, and neutral) in the task conditions during the stimulus presentation in Experiment 1. The horizontal axis indicates the time (s), while the vertical axis indicates the grand-averaged change in pupil dilation from baseline (−200 ms to 0 ms).

**Fig 5:**
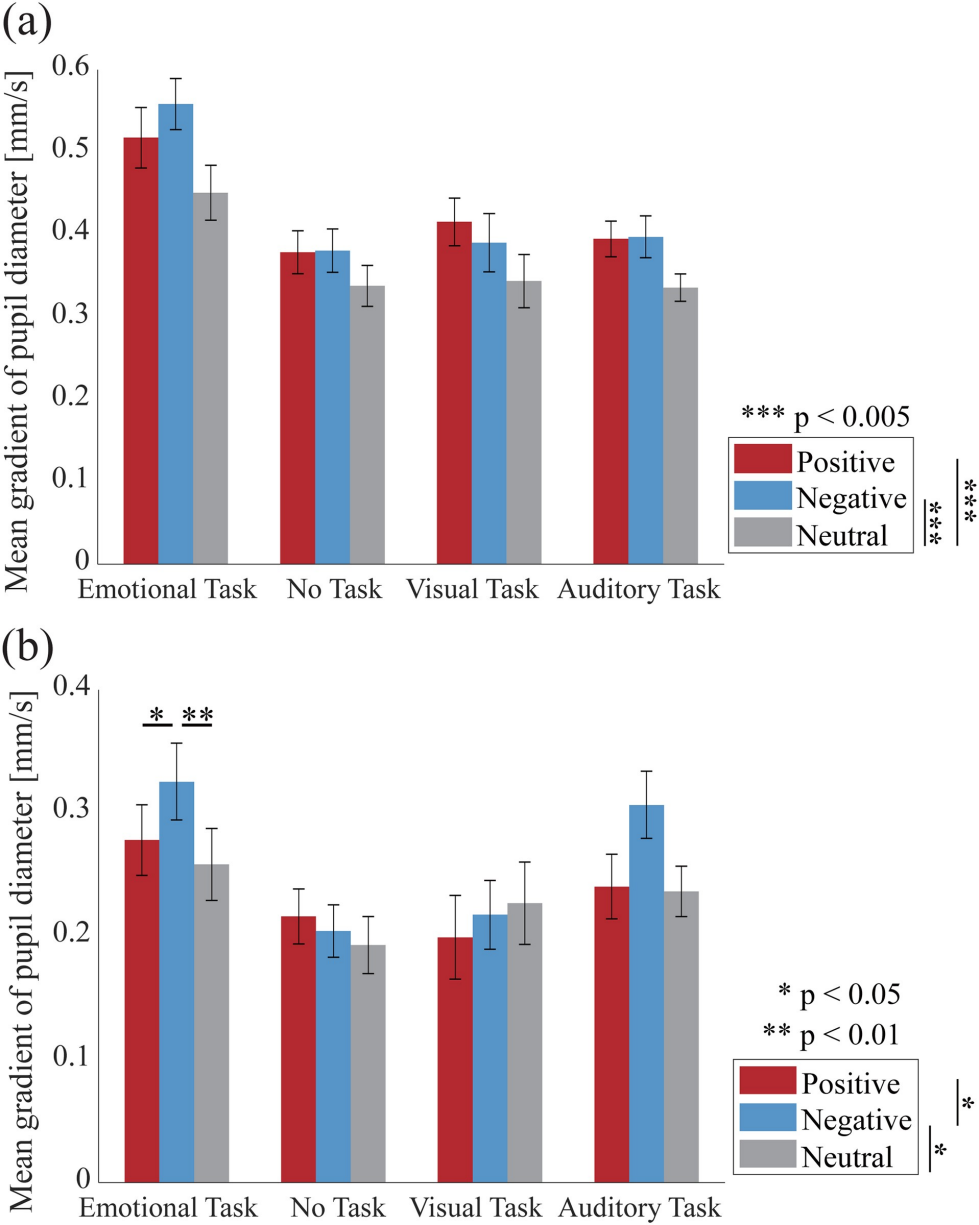
The velocity of pupil dilation. **(a)** Gradient values of the pupillary response to the IAPS pictures for each condition in Experiment 1. **(b)** Gradient values of the pupillary response to the IADS sounds for each condition in Experiment 2. Error bars show standard error of the mean across the participants. Asterisks in (b) indicate a significant difference based on multiple comparisons for the interaction between the stimulus condition and the task condition; * *p* < 0.05, ** *p* < 0.01, *** *p* < 0.005.

Figs 3 and 4 show the grand-average time course of changes in the pupil dilation during stimulus presentation (6,000 ms) in each task condition for Experiment 1 and 2. Fig 5a shows the gradient values of the pupillary response to the IAPS pictures for each condition in Experiment 1. ANOVAs revealed a significant main effect of the stimulus condition [*F*(2, 46) = 10.660; *p* < 0.001; *partial η*^*2*^ = 0.317; *BF*_*10*_ = 2.170 × 10^3^]. The planned comparisons revealed that the gradient values for the negative and positive pictures were significantly greater than those for the neutral pictures (*p* = 0.002 and *p* = 0.002 for negative and positive pictures, respectively). However, the comparison between the positive and negative pictures revealed no significant difference (*p* = 0.735).

A significant main effect of the task condition was observed for the gradient values [*F*(3, 69) = 15.304; *p* < 0.001; *partial η*^*2*^ = 0.400; *BF*_*10*_ = 4.804 × 10^12^]. The planned comparisons revealed that the gradient value for the emotional task was significantly greater than that for the no task, the visual task, and the auditory task (*p* = 0.001, *p* = 0.001, *p* < 0.001, respectively). However, there was no significant interaction effect between the stimulus condition and the task condition [*F*(6, 138) = 1.562; *p* = 0.163; *partial η*^*2*^ = 0.064]. In addition, we analyzed the gradient values with a Bayesian repeated measures ANOVA. We found a *BF*_*10*_ smaller than 0.33 in the interaction between emotion and task (*BF*_*10*_ = 0.031). Thus, we have evidence in support of the null hypothesis that there is no interaction between emotion and task. These results indicate that the positive and negative pictures triggered faster pupil dilation compared to that for the neutral pictures in all task conditions.

Fig 5b shows the gradient values of the pupillary response to the IADS sounds for each condition in Experiment 2. ANOVA revealed a significant main effect of the stimulus condition, although Bayes factors was providing weak evidence against this effect [*F*(2, 46) = 5.595; *p* = 0.007; *partial η*^*2*^ = 0.196; *BF*_*10*_ = 0.465]. The planned comparisons revealed that the gradient values for the negative sounds were significantly greater than those for the positive and neutral sounds (*p* = 0.017, *p* = 0.017, respectively). However, there was no significant difference between the positive and neutral sounds (*p* = 0.673).

A significant main effect of the task condition was observed for the gradient values [*F*(3, 69) = 4.990; *p* = 0.003; *partial η*^*2*^ = 0.178; *BF*_*10*_ = 3.760 × 10^3^]. The planned comparisons revealed that the gradient value for the emotional task was significantly greater than that for the no task and the visual task (*p* = 0.015 and *p* = 0.016 for the no task and the visual task, respectively). Moreover, the gradient value for the auditory task was significantly greater than that for the no task (*p* = 0.015).

There was a trend towards a significant interaction between the stimulus condition and the task condition, however, Bayes factors providing strong evidence against this interaction [*F*(6, 138) = 1.823; *p* = 0.099; *partial η*^*2*^ = 0.073; *BF*_*10*_ = 0.101]. We observed simple main effects of the task condition on the negative sounds [*F*(3, 69) = 6.653; *p* = 0.001; *partial η*^*2*^ = 0.224] and a trend for the positive sounds [*F*(3, 69) = 2.458; *p* = 0.070; *partial η*^*2*^ = 0.097]. Further analyses revealed that the gradient value for the negative sounds was significantly greater for the emotional task than that for the no task and the visual task (*p* = 0.007 and *p* = 0.002, for the no task and the visual task, respectively). The gradient value for the negative sounds was also significantly greater for the auditory task than that for the no task (*p* = 0.002). The gradient value for the positive sounds trended towards being significantly greater for the emotional task than for the visual task (*p* = 0.074). We observed simple main effects of the stimulus condition on the emotional task [*F*(2, 46) = 5.460; *p* = 0.008; *partial η*^*2*^ = 0.192] and the auditory task [*F*(2, 46) = 4.026; *p* = 0.025; *partial η*^*2*^ = 0.149]. Further analyses revealed that the gradient value for the emotional task was significantly greater for the negative sounds than that for the positive sounds and the neutral sounds (*p* = 0.048, *p* = 0.007, respectively). The gradient value for the auditory task trended towards being significantly greater for the negative sounds than for neutral and positive sounds (*p* = 0.053 and *p* = 0.053 for the neutral and positive sounds, respectively). These results suggest that in the emotional and auditory tasks, the negative sounds triggered a faster pupil dilation compared to that for the positive and neutral sounds.

In addition, three-way ANOVAs with repeated measures were performed using the pupillary gradient in 3 emotion conditions and the 4 task conditions, as the within-subjects factor, and 2 modality conditions (Vision vs. Audition) as the between-subjects factor. However, there was no significant interaction effect between the stimulus condition, the task condition and modality condition [*F*(5.33, 245.15) = 1.430; *p* = 0.221; *partial η*^*2*^ = 0.003; *BF*_*10*_ = 0.0345].

## Discussion

In the present study, we investigated the relationship of attentional states to emotional unimodal stimuli (the IAPS pictures or the IADS sounds) and emotional responses using pupillometry in two experiments. Results for the velocity of pupillary responses showed that the emotional responses to visual stimuli were elicited regardless of the task (any attentional state), while the emotional responses to auditory stimuli were elicited only when the participants attended to the auditory modality. Thus, our results point to different properties of emotional responses for visual and auditory stimuli at least in the point that there is no attentional effect to emotional responses to visual stimuli.

In Experiment 1, there was no significant interaction for the velocity of the pupil dilation between the stimulus condition and the task condition; compared to the neutral pictures, both the positive and negative pictures elicited a larger pupil dilation in all task conditions. The results for the emotional task and the no task in Experiment 1 coincided with findings from the previous study indicating that the pupil diameter dilated more to the emotional pictures than to the neutral pictures regardless of the participants’ mode of viewing [22]. Schupp et al. reported an interference with the selective emotional processing when visual attentional resources are allocated for a visual target (horizontal or vertical lines) superimposed on the IAPS pictures [25]. However, in our experiment, the target dot or sound during an emotional stimulus presentation in both visual and auditory tasks was not presented in all trials analyzed for the pupillary response. Therefore, we conjectured that the emotional responses to the IAPS pictures were elicited in all task conditions because the visual target (e.g., target dot) to divert attention did not exist, which is not dissimilar to the reports of Schupp et al. Moreover, our pupillometry results for the auditory task in Experiment 1 revealed that the velocity of the pupil dilation when the participants attended to the emotional pictures was faster compared to that for the neutral pictures. Schupp et al. reported that the processing of the emotional pictures was not modulated by the additional auditory detection task of increasing complexity [26], suggesting that the emotion processing in the visual domain is not affected by the task demands in the auditory modality [3]. Thus, the processing of the emotional pictures in Experiment 1 was unlikely to be disrupted by the auditory task because visual and auditory modalities use independent attentional resources.

In Experiment 2, there was a trend towards a significant interaction between the stimulus condition and the task condition for the velocity of the pupil dilation, suggesting that the velocity of the pupil dilation for the negative sounds in the emotional and auditory tasks may have been faster than that for the positive and neutral sounds. Although our results are consistent with the previous findings [17] in that pupillary response to the negative sounds was larger than that to the neutral sounds, the results for the pupillary response to the positive sounds were different from the previous findings. This difference may be due to the results of the subjective rating to the IADS sounds in the emotional task, whereby negative emotions were elicited more than positive and neutral emotions. Therefore, the velocity of the pupil dilation for the negative sounds in the emotional and auditory tasks may have been faster than that for the positive sounds because the emotional evaluation of the positive sounds was lower than for the negative sounds [24]. The no task and the visual task in Experiment 2 did not need as much auditory attention during the stimulus presentation compared to the emotional task and the auditory task, indicating that the emotional responses of the pupillary change in Experiment 2 were elicited only in the conditions where the participants attended to auditory modalities. Thus, the emotional responses to the sounds in the no task and the visual task may have been suppressed because attention was paid to the fixation point of a different modality rather than to the emotional stimuli, in an agreement with the previous study [26].

The dissimilarity of the pupillary responses between Experiment 1 and Experiment 2 included the following: whereas emotional responses to visual stimuli were elicited in all task conditions, emotional responses to auditory stimuli were elicited only when attention was paid to the auditory modality during the stimulus presentation (emotional task, auditory task). Previous studies on unconscious emotional processing of visual stimuli have demonstrated that emotional responses can be elicited by subliminal perception [41–43]. In contrast, Lähteenmäki et al. reported that the affective auditory processing is dependent on awareness, and emotional responses were not elicited during the nonconscious auditory processing [44]. In our experiments, there was no difference in emotional responses between the visual and auditory stimuli when attention was paid to the emotional stimuli (Experiment 1: emotional task & visual task, Experiment 2: emotional task & auditory task). However, in the conditions in which the participants did not need to attend to emotional stimuli (Experiment 1: no task & auditory task, Experiment 2: no task & visual task), the pupillary response to emotional visual stimuli was more dilated compared to that of the neutral pictures, whereas the pupillary response to emotional auditory stimuli did not dilate. We, therefore, propose a different feature of emotional responses to visual and auditory stimuli, whereby unconscious emotional responses to visual stimuli are elicited whereas unconscious emotional responses to auditory stimuli are not evoked (or minimally evoked).

Taken together, the current data of pupillary responses reveal emotional responses depend on attentional allocation to visual and auditory stimuli. Emotional responses to visual stimuli were evoked in all attentional states, whereas emotional responses to auditory stimuli were only evoked in cases where the participants attended to the auditory modality. However, there are some limitations in this study considering most effects that are not a large effect size, and non-significant interaction between the stimulus condition, the task condition, and modality condition, which undermine our conclusion of asymmetrical characteristics of emotional responses to pictures and sounds. In this study, we used an erotic stimulus, which might cause a gender difference in emotional response, and we could not strictly control the emotional valence of the stimuli between two modalities because we analyzed pupillary response according to the classification of the emotional task. In addition, the visual stimuli in Experiment 1 were static images, whereas the auditory stimuli in Experiment 2 were dynamic sounds. This difference might influence the eye movement during each trial, which is possible to be a potential contaminant for pupillary responses. Further studies using sufficiently controlled stimuli (video and sound) between two modalities are needed in order to investigate the difference in temporal processing between the two modalities. Moreover, the partial overlap of the participants between Experiment 1 and 2 more complicated the comparison of pupillary responses between the experiments.

Our findings suggest that differences in emotional responses to visual and auditory stimuli must be considered in any research on cognitive emotion regulation. This will be conducive to the establishment of new methods of cognitive emotional regulation, whereby emotional responses elicited by one sensory modality are prompted or suppressed by stimuli from another sensory modality.

## Supporting information

S1 Data Participant data sets are available from github (https://github.com/s143356/Experimental_data/tree/master/Project_01). (DOCX)

S1 Text IAPS numbers in Experiment 1 and IADS numbers in Experiment 2. (DOCX)
