# Supplementary material for: Asymmetrical characteristics of emotional responses to pictures and sounds: Evidence from pupillometry
Source: PLoS One. 2020 Apr 6;15(4):e0230775. doi: 10.1371/journal.pone.0230775 (PMC7135059; doi:10.1371/journal.pone.0230775)
Supplement: S1 Data — (DOCX) [file pone.0230775.s001.docx]

**Participant data sets are available from github (**<https://github.com/s143356/Experimental_data/tree/master/Project_01>**).**
