# Supplementary material for: Asymmetrical characteristics of emotional responses to pictures and sounds: Evidence from pupillometry
Source: PLoS One. 2020 Apr 6;15(4):e0230775. doi: 10.1371/journal.pone.0230775 (PMC7135059; doi:10.1371/journal.pone.0230775)
Supplement: S1 Text — (DOCX) [file pone.0230775.s002.docx]

***・IAPS pictures***

The IAPS pictures (Lang et al., 2008) used in this study were: Positive: 4220, 4250, 4290, 4599, 4643, 4659, 4687, 5629, 7280, 8030, 8034, 8170, 8185, 8193, 8300, 8370, 8461, 8470, 8501, 8549; Negative: 1019, 1120, 1220, 1274, 2683, 2688, 2981, 3150, 3350, 5971, 6021, 6230, 6242, 6510, 8485, 9250, 9429, 9622, 9902, 9921; Neutral: 1675, 1726, 2357, 2372, 2512, 2514, 2595, 2635, 5395, 7036, 7037, 7043, 7207, 7211, 7242, 7487, 7493, 7640, 8475, 9402.

***・IADS sounds***

The IADS sounds (Bradley & Lang, 2007) used in this study were: Positive: 110, 200, 202, 220, 224, 230, 311, 352, 353, 360, 365, 367, 717, 726, 811, 813, 815, 816, 817, 820; Negative: 105, 116, 241, 242, 244, 250, 280, 283, 289, 295, 296, 423, 501, 600, 611, 626, 703, 711, 713, 730; Neutral: 104, 170, 171, 246, 262, 322, 373, 376, 376, 377, 382, 627, 698, 700, 701, 705, 708, 720, 722, 723.
